# Sexual and gender minority individuals report higher rates of abuse and more severe eating disorder symptoms than cisgender heterosexual individuals at admission to eating disorder treatment

**DOI:** 10.1002/eat.23257

**Published:** 2020-03-13

**Authors:** Janell L. Mensinger, Janeway L. Granche, Shelbi A. Cox, Jennifer R. Henretty

**Affiliations:** ^1^ M. Louise Fitzpatrick College of Nursing Villanova University Villanova Pennsylvania USA; ^2^ Department of Epidemiology and Biostatistics, Dornsife School of Public Health Drexel University Philadelphia Pennsylvania USA; ^3^ Center For Discovery Discovery Behavioral Health Los Alamitos California USA

**Keywords:** abuse, eating disorder treatment, eating disorder outcome, higher levels‐of‐care, Minority Stress Theory, sexual/gender minority, transgender, trauma

## Abstract

Eating disorders (EDs) occur at higher rates among sexual/gender minorities (SGMs). We currently know little about the risk factor profile of SGMs entering ED specialty care.

**Objective:**

To (a) compare history of abuse‐related risk in SGMs to cisgender heterosexuals (CHs) when entering treatment, (b) determine if SGMs enter and exit treatment with more severe ED symptoms than CHs, and (c) determine if SGMs have different rates of improvement in ED symptoms during treatment compared to CHs.

**Method:**

We analyzed data from 2,818 individuals treated at a large, US‐based, ED center, 471 (17%) of whom identified as SGM. Objective 1 was tested using logistic regression and Objectives 2 and 3 used mixed‐effects models.

**Results:**

SGMs had higher prevalence of sexual abuse (OR = 2.10, 95% CI = 1.71, 2.58), other trauma (e.g., verbal/physical/emotional abuse; OR = 2.07, 95% CI = 1.68, 2.54), and bullying (OR = 2.13, 95% CI = 1.73, 2.62) histories. SGMs had higher global EDE‐Q scores than CHs at admission (*γ* = 0.42, SE = 0.08, *p* < .001) but improved faster early in treatment (*γ* = 0.316, SE = 0.12, *p* = .008). By discharge, EDE‐Q scores did not differ between SGMs and CHs.

**Discussion:**

Our main hypothesis of greater abuse histories among SGMs was supported and could be one explanation of their more severe ED symptoms at treatment admission compared to CHs. In addition, elevated symptom severity in SGMs at admission coincides with greater delay between ED onset and treatment initiation among SGMs—possibly a consequence of difficulties with ED recognition in SGMs by healthcare providers. We recommend increased training for providers on identifying EDs in SGMs to reduce barriers to early intervention.

## 1 INTRODUCTION

Research shows that sexual and gender minority individuals (hereon referred to as SGMs) (i.e., lesbian, gay, bisexual, transgender, or other noncisgender or nonheterosexual identities) have significantly higher rates of numerous mental and physical health conditions compared to their cisgender heterosexual peers (e.g., Branstrom, Hatzenbuehler, & Pachankis, 2016; Gonzales, Przedworski, & Henning‐Smith, 2016; King et al., 2008; Operario et al., 2015). Eating disorders (EDs) are among the many conditions that show elevated rates in this population (Austin, Nelson, Birkett, Calzo, & Everett, 2013; French, Story, Remafedi, Resnick, & Blum, 1996; McClain & Peebles, 2016; Watson, Adjei, Saewyc, Homma, & Goodenow, 2017). Although this disparity is most consistently found in SGM men (Calzo, Blashill, Brown, & Argenal, 2017; Diemer, Grant, Munn‐Chernoff, Patterson, & Duncan, 2015; Morrison, Morrison, & Sager, 2004), a systematic review of the literature suggested that sexual minority women also tend to have higher rates of ED diagnoses and are at greater risk for engaging in disordered eating behaviors than cisgender heterosexual (CH) women, despite having less body dissatisfaction and drive for thinness (among the two most robust ED risk factors) than CH women and SGM men (Bergeron & Senn, 1998; Meneguzzo et al., 2018). Accordingly, researchers have studied several theories, including Minority Stress Theory (Meyer, 2003) to aid in understanding reasons for these differences (e.g., Brewster, Velez, Breslow, & Geiger, 2019; Calzo et al., 2017).

Minority Stress Theory posits that stigmatization and social exclusion are ongoing chronic stressors contributing to dysregulation of multiple organ systems in the body and ultimately causing the higher rates of chronic diseases and poorer health outcomes found in marginalized and oppressed populations (e.g., Hatzenbuehler & McLaughlin, 2014; Stuber, Meyer, & Link, 2008). The experience of persistent discrimination and micro‐aggressions often results in a vulnerability that places individuals at higher risk for victimization in the forms of bullying, abuse (sexual, physical, and emotional), and other forms of violence (e.g., Balsam, Rothblum, & Beauchaine, 2005; Corliss, Cochran, & Mays, 2002; Kann et al., 2011). The minority stress literature has led to the conceptualization of stigma as a “fundamental cause” of inequalities in population health (Hatzenbuehler, Phelan, & Link, 2013). Furthermore, in their systematic review, Alencar Albuquerque et al. (2016) theorized that SGMs are less likely to seek treatment in part due to internalized anti‐SGM bias and shame. For these reasons, investigations of treatment response in oppressed and marginalized groups—such as SGMs—must be a high priority for public health research.

Despite recent evidence showing that SGM individuals have higher rates of lifetime ED diagnoses as well as unhealthy weight control behaviors than their CH peers (Kamody, Grilo, & Udo, 2019; Meneguzzo et al., 2018), risk factors and pathways leading to the disorder are often different for these groups (Duffy, Henkel, & Earnshaw, 2016; Engeln‐Maddox, Miller, & Doyle, 2011; Wang & Borders, 2017; Watson, Grotewiel, Farrell, Marshik, & Schneider, 2015). For instance, research has shown gender dysphoria contributes to EDs in transgender individuals (Duffy et al., 2016). In sexual minority men, Wiseman and Moradi (2010) found experiences of sexual objectification and childhood homophobic bullying were associated with disordered eating attitudes and behaviors. Similarly, Feldman and Meyer (2007) identified higher rates of EDs in SGM men who had experiences of childhood abuse compared to those without abuse histories. Thus, it is important to elucidate whether trauma‐related risk factors (such as having experienced bullying and/or sexual, physical, or emotional abuse) are more prevalent in SGM compared to CH individuals who present for higher levels‐of‐care at an ED treatment center. A better understanding will aid in developing and tailoring SGM‐specific screening tools and interventions, which, to our knowledge, are lacking.

In accordance with Minority Stress Theory, this study aims to determine if, compared to CH patients: (a) SGM patients have a greater prevalence of abuse history (including bullying, sexual abuse, and other forms of physical and emotional trauma) when they present to ED treatment; (b) SGM patients enter and exit treatment with more severe ED symptomatology scores; and (c) SGM patients have different trajectories of improvement in ED symptoms over the course of their treatment episode. Specifically, we will test the following Hypotheses: (1) SGMs will present to treatment with higher rates of abuse (bullying, sexual, physical/emotional/verbal) than CH peers, (2) SGMs will (a) enter and (b) exit treatment with more severe ED symptoms than CH peers, and (3) SGMs will respond to treatment (in terms of ED symptom reduction) more slowly than their CH peers.

## 2 METHOD

### 2.1 Participants and setting

This retrospective, longitudinal cohort study utilized a de‐identified dataset of 2,818 participants with diagnosed EDs entering higher levels‐of‐care in a large, US‐based, ED treatment center. The majority (*n* = 2,049) entered at one of the residential treatment centers, 459 entered a partial hospital program, and 310 entered an intensive outpatient program. Eligibility for higher level‐of‐care treatment was determined by the American Psychiatric Association's Practice Guidelines for the Treatment of Patients with Eating Disorders (Yager et al., 2014) and medical necessity criteria established by third‐party payors. Ninety percent of the Center's population has their treatment covered by private insurance, 8% is self‐pay, and 2% is scholarship.

A majority (95%, *n* = 2,667) of the sample identified as female and 5% (*n* = 151) as male. Of the total sample, 17% (*n* = 471) identified as SGM, 7% (*n* = 189) as unsure of their sexual orientation, and 77% (*n* = 2,153) as both cisgender and heterosexual (SGM category definitions are explained more explicitly under Section 2.3). Five female patients did not give a sexual orientation and were therefore coded as missing on the SGM status variable. Most of the sample identified as White non‐Hispanic (78%, *n* = 2,112), 12% (*n* = 327) as Latinx, 4% (*n* = 123) as Asian or Pacific Islander, 2% (*n* = 49) as African American or Black, 1% (*n =* 15) as American Indian or Native Alaskan, and 3% (*n* = 95) as biracial. The remaining 3.4% (*n* = 97) did not respond to the racial/ethnic identity question. For additional sample characteristics, see Table 1.

**Table 1:** Sample characteristics by sexual/gender minority status

	Sexual/gender minority	Unsure	Cisgender heterosexual	Total	
Characteristic/variable	*n* = 471 (17%)	*n* = 189 (7%)	*n* = 2,153 (77%)	*N* = 2,813^a^ (100%)	*p* b
*Gender (N = 2,813)* c	*n* (%)	*n* (%)	*n* (%)	*n* (%)	.03
Female	443 (94)	171 (90)	2,048 (95)	2,662 (95)	
Male	28 (6)	18 (10)	105 (5)	151 (5)	
*Age group (N = 2,813)*					<.001
18 and younger	280 (59)	149 (79)	1203 (56)	1,632 (58)	
19 and older	191 (41)	40 (21)	950 (44)	1181 (42)	
*Race/ethnicity (N = 2,717)*					.11
White Non‐Hispanic	336 (74)	137 (76)	1,637 (79)	2,110 (78)	
Hispanic	61 (13)	28 (15)	238 (11)	327 (12)	
Asian/Pacific Islander	19 (4)	9 (5)	93 (4)	121 (4)	
Black	9 (2)	1 (1)	39 (2)	49 (2)	
Biracial	26 (6)	6 (3)	63 (3)	95 (3)	
Native American/Alaskan Native	4 (1)	‐‐	11 (1)	15 (1)	
*History of sexual abuse (N = 2,808)*					<.001
Yes	202 (43)	56 (30)	568 (26)	826 (29)	
No	268 (57)	132 (70)	1,582 (74)	1,982 (71)	
*History of bullying (N = 2,806)*					<.001
Yes	302 (64)	112 (60)	980 (46)	1,394 (50)	
No	169 (36)	76 (40)	1,167 (54)	1,412 (50)	
*History of other trauma* d *(N = 2,812)*					<.001
Yes	302 (64)	88 (47)	998 (46)	1,388 (49)	
No	169 (36)	100 (53)	1,155 (54)	1,424 (51)	
*Initial level‐of‐care (N = 2,813)*					.002
Residential treatment center	340 (72)	157 (83)	1,550 (72)	2,047 (73)	
Partial hospital program	92 (20)	18 (10)	349 (16)	459 (16)	
Intensive outpatient program	39 (8)	14 (7)	254 (12)	307 (11)	
*ED diagnosis (N = 2809)*					<.001
Anorexia nervosa‐restricting	164 (35)	87 (46)	995 (46)	1,246 (44)	
Anorexia nervosa‐w/purging	103 (22)	41 (22)	353 (16)	497 (18)	
Bulimia nervosa	108 (23)	26 (14)	398 (19)	532 (19)	
Binge eating disorder	33 (7)	8 (4)	157 (7)	198 (7)	
OSFED	62 (13)	27 (14)	247 (11)	336 (12)	
*Prior ED treatment (N = 2,811)*					<.001
Yes	346 (74)	151 (80)	1,749 (81)	2,246 (80)	
No	124 (26)	38 (20)	403 (19)	565 (20)	
Characteristic/variable	Mean (SD)/median (IQR)	Mean (SD)/median (IQR)	Mean (SD)/median (IQR)	Mean (SD)/median (IQR)^a^	*p* b
Months since ED onset (*N* = 2,800)^e^	48 (24‐108)	24 (12‐60)	36 (12‐108)	36 (12‐106)	<.001
Age in years (*N* = 2,813)	19.7 (7.48)	17.1 (5.54)	21.3 (10.01)	20.7 (9.46)	<.001
Intake BMI (*N* = 2,813)	23.2 (7.85)	20.6 (6.18)	21.4 (7.25)	21.7 (7.32)	<.001
Intake EDE‐Q score (*N =* 2,495)	4.00 (1.45)	3.60 (1.56)	3.57 (1.63)	3.64 (1.61)	<.001

Abbreviations. BMI, body mass index; ED, eating disorder; EDE‐Q, Eating Disorder Examination Questionnaire; OSFED, other specified feeding or eating disorder.

a Five participants were missing sexual orientation data.

b *p*‐value represents omnibus F‐statistics on mean/median comparisons or Chi‐square test of independence; to reach statistical significance using a Bonferroni‐adjusted *p*‐value (based on all 13 factors tested) *p*s must be <.004.

c Only male and female genders are presented because no patients self‐identified as nonbinary.

d Includes forms of abuse other than sexual abuse and bullying (e.g., physical, verbal, and emotional).

e The extreme right skew of this variable required log transformation for use in the predictive model. We are presenting median and interquartile ranges here to enhance interpretation and correct for the distribution skew.

### 2.2 Materials and procedure

**Figure 1:**
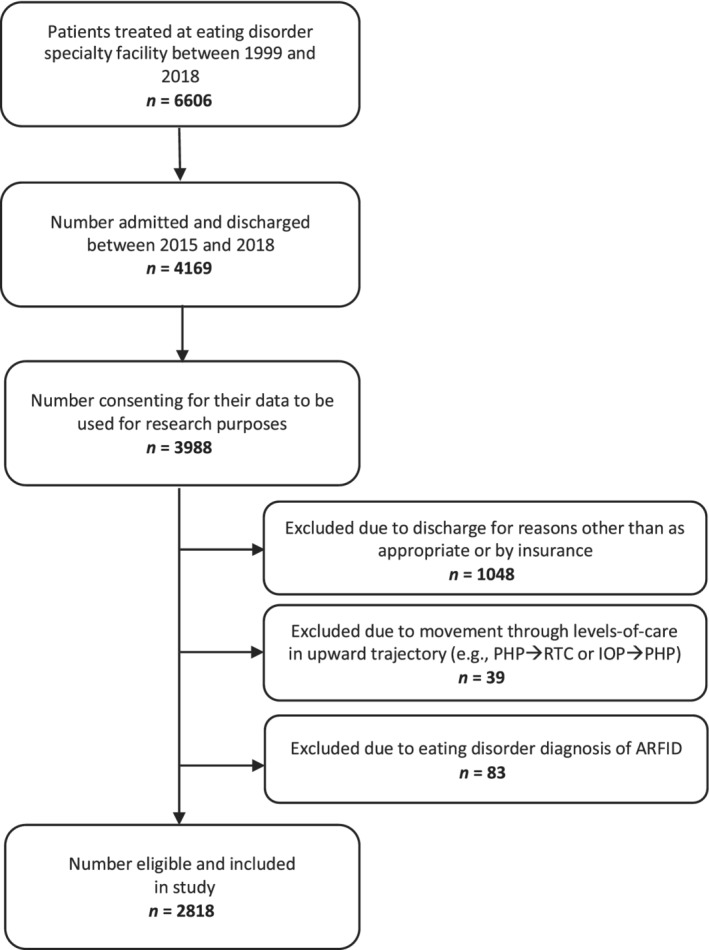
STROBE flow diagram. Abbreviations: ARFID, avoidant restrictive food intake disorder; IOP, intensive outpatient program; PHP, partial hospitalization program; RTC, residential treatment center; STROBE, Strengthening the Reporting of Observational Studies in Epidemiology

**Figure 2:**
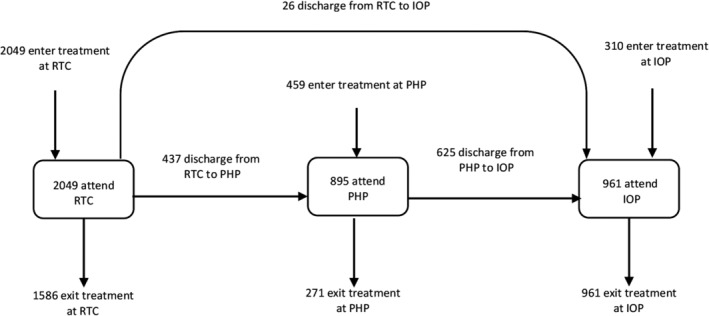
Flow of eating disorder patients through levels‐of‐care. Abbreviations: IOP, intensive outpatient program; PHP, partial hospitalization program; RTC, residential treatment center

Participants were eligible for study inclusion if they: (a) admitted and discharged between 2015 and 2018, (b) consented for their data to be used for research purposes, (c) were discharged in accordance with the treatment plan or by insurance determination (i.e., discharged into a lower level‐of‐care), and (d) had an ED diagnosis other than avoidant/restrictive food intake disorder (ARFID) (see Figure 1 for the STROBE flow diagram). Patients with ARFID (*n* = 83) were excluded due to research showing that our main outcome measure (i.e., global scores on the Eating Disorder Examination‐Questionnaire, EDE‐Q) does not sufficiently capture ARFID symptomology (Cooney, Lieberman, Guimond, & Katzman, 2018). During treatment, participants were followed through the program as they moved through higher levels‐of‐care in a descending trajectory (e.g., residential treatment to partial hospitalization to intensive outpatient, or partial hospitalization to intensive outpatient). Data were recorded at admission and discharge from each level‐of‐care. To avoid duplication of individuals who may have had more than one treatment episode in the allotted timeframe, only participants' most complete treatment episode was retained to facilitate the longitudinal modeling process. If two or more episodes contained admission and discharge data from only one level‐of‐care, only the first treatment episode was retained. The final dataset contained 7,456 observations, with between two and six data points for each patient. See Figure 2 for details of the sample's movement through the higher level‐of‐care system. Ethical review of the study was provided by the Drexel University Institutional Review Board.

### 2.3 Study measures

Global EDE‐Q scores (Fairburn, 2009) were used as the primary outcome variable. The EDE‐Q is a well‐validated and reliable tool for clinical samples (e.g., Binford, Le Grande, & Jellar, 2005; Dahlgren, Stedal, & Rø, 2017) and has been successfully used to measure symptom change following a treatment intervention (e.g., Sysko, Walsh, & Fairburn, 2005). Specifically, the EDE‐Q measures cognitive and behavioral aspects of ED symptomatology over the prior 28 days using a scale from 0 (*no days*) to 6 (*every day*). Patients were asked to complete the EDE‐Q at admission and discharge from each level‐of‐care.

Our main exposure variable—SGM status—was created using information gathered during structured clinical intake interviews via licensed clinicians (or clinicians overseen by licensed clinicians). Clinicians asked patients their sexual orientation—defined as straight/heterosexual, lesbian/gay, bisexual, unsure, or other—and, whether they identified as transgender. Patients were also asked their gender‐identity (as opposed to sex assigned at birth). The electronic medical record (EMR) allowed for answers of female, male, or blank, with a text field that could be filled in with nonbinary genders. Gender‐identity and sexual orientation were combined to form a variable with three categories for SGM status: (a) those who identified as cisgender and heterosexual; (b) SGMs, who identified as transgender and/or as lesbian/gay, bisexual, or other; and (c) unsure individuals, who were cisgender but identified their sexual orientation as “unsure.” Table 1 shows the breakdown of gender identification by SGM status. Notably, all patients identified themselves as either female or male, as opposed to nonbinary; thus, gender‐identity is presented in only two categories.

History of abuse and bullying were also assessed during the structured intake interviews. Consistent with the guidelines and recommendations for screening for abuse found in the Department of Health and Human Services (SAMSHA/CSAT branches) prepared volume on *Substance Abuse Treatment for Persons with Child Abuse and Neglect Issues* (Center for Substance Abuse Treatment, 2000), questions posed to operationalize abuse history were as follows: “Did you ever experience any of the following forms of abuse?” The clinician inquired about sexual, physical, emotional, verbal, spiritual, and mental abuse. A subsequent question asked the patient: “Did you ever experience trauma or abuse not described above?” (though 100% of the responses to this question were negative). A third question asked: “Have you ever been bullied or threatened?” Response options included “*yes*” or “*no*” in addition to a free text box for the therapist to add descriptive notes as necessary for contextualizing the responses (therapist notes were not incorporated into the deidentified data used in the present article). For the purposes of our analyses, variables were coded as yes/no to represent a history of: (a) sexual abuse, (b) bullying, and (c) other trauma, which compiled any form of the remaining types of abuse experienced. The components of the trauma variable were internally consistent with a Cronbach's alpha coefficient of .79. Validity of the separate trauma composite was verified with a principal component analysis, which suggested a one‐factor solution.

Two a priori specified covariates were also drawn from the structured clinical intake interviews: months‐since‐ED‐onset and prior history of ED treatment in a higher level‐of‐care (yes/no). To characterize the study sample, we also extracted the following variables from the EMR: patients' current ED diagnoses, weight and height (from which body mass index—BMI—was calculated as weight in kilograms divided by height in meters^2^), race/ethnicity, and age. To account for time (the primary predictor variable for testing Hypothesis 3) and model the trajectory of change in EDE‐Q scores over the course of care, we measured “days in treatment” as the number of days since admission—beginning with 0 to mark the initial admission day—and the exact day of discharge and admission into the next lower level‐of‐care to numerically mark each following timepoint.

### 2.4 Statistical analysis

Data were analyzed in SPSS (Version 25, Armonk, NY: IBM Corp.) and SAS (Version 9.4, Cary, NC: SAS Institute Inc.). To check for outliers and examine data distributions, we completed exploratory data analyses—including measures of central tendency, skewness and kurtosis, histograms, stem‐and‐leaf, and box‐and‐whisker plots on the outcome and predictor variables. A log transform was applied to the months‐since‐ED‐onset variable to correct for an extreme right skew. All other variables adequately met distribution assumptions. To reduce problems with multicollinearity when applying confounder‐adjustments to the model testing Hypotheses 2 and 3, we categorized the age variable (<19 or not), which prior to doing so was highly collinear with months‐since‐ED‐onset (*r* > .80).

We used logistic regression analysis with Bonferroni adjustments to provide a conservative correction for multiple comparisons to test Hypothesis 1 (*α* = .017 with 3 comparisons). To investigate Hypotheses 2a, 2b, and 3, we fit curvilinear mixed models with random intercepts and multiple slopes. The final model form is composed of a linear function of time (measured in total days between initial admission and final discharge) and an inverse function of time. The inverse time component dominates the model during the initial phase of treatment, and thus corresponds to rate of acceleration or “initial improvement,” while the linear time function corresponds to the rate of change in the later phase of treatment. Model form was chosen after fitting a series of Empirical Bayes plots and unconditional growth models of different combinations of time functions to determine the best average change trajectory to represent the data. We used likelihood ratio tests and Akaike Information Criterion to choose the best model fit for determination of slope functions. Hypotheses 2a, 2b, and 3 were tested by entering SGM status as a fixed effect predicting model intercepts (initial admission EDE‐Q score) and slopes (rate of change in EDE‐Q scores over the course of treatment). We present the models progressively in a traditional growth curve modeling framework (Singer & Willet, 2003), which are analogous to mixed‐effects models for longitudinal data (e.g., Bryk & Raudenbush, 2002). The initial model represents an “unconditional means” model with only a random intercept fit, and Model 2 is the “unconditional growth” model with random intercepts and slopes. We included patients’ entry level‐of‐care in the unconditional models to account for the sample structure. Model 3 tests SGM status as fixed effects predicting the intercept and slope coefficients. Initial admission and exit global EDE‐Q scores were estimated at Day 0 and Day 119 (the cohort's median length of stay for those who received all levels‐of‐care) using contrast statements and estimated marginal means (*EMMs*). Model 4 was a confounder‐adjusted model controlling for the three abuse variables (bullying, sexual abuse, and other trauma), months‐since‐ED‐onset, prior ED treatment, age at intake (<19 years or not), intake BMI, and gender‐identity. Despite low power, gender‐identity was entered as a potential moderator of the effects of SGM status in the confounder‐adjusted model given the literature showing men tend to score significantly lower on average than women on the EDE‐Q (Hilbert, de Zwaan, & Braehler, 2012). All covariates were fixed effects predicting the Level‐2 coefficients of mean initial admission status and both mean rates of change. Model assumptions were tested using plots of predicted‐against‐observed values and examining the distributions of the intercepts and slopes. Mixed‐modeling approaches invoke maximum likelihood estimation to handle missing data; this is robust to bias under the assumption of Missing at Random, meaning that data are missing as a function of the observed but not unobserved data (Little & Rubin, 1987). Using mixed modeling approaches, all cases having at least one observation on the outcome (as did all cases in this dataset) are included in the analysis (Laird, 1988). However, cases with a greater number of repeated observations are weighted more heavily when estimating parameters. Given missingness on predictor variables was minimal (<0.5%), over 99% of the sample was included in all of the analyses shown thus making sensitivity models for missing data unwarranted.

## 3 RESULTS

Supporting Hypothesis 1, SGM patients, compared to CH patients, were more likely to have a history of sexual abuse (OR = 2.10, 95% CI = 1.71, 2.58), bullying (OR = 2.13, 95% CI = 1.73, 2.62), and other trauma (OR = 2.07, 95% CI = 1.68, 2.54). After a Bonferroni correction for multiple comparisons, *p*‐values remained significant (all *p*s < .001).

In support of Hypothesis 2a, the model estimated that SGM patients entered treatment with significantly higher EDE‐Q scores than their CH peers, estimated marginal mean difference, (*EMM* *Δ* = 0.422, 95% CI = 0.255, 0.588). However, upon exiting treatment (Hypothesis 2b), SGM patients' EDE‐Q scores were not significantly different than their CH peers (*EMM* *Δ* = 0.110, 95% CI = −0.173, 0.392). Tests of Hypothesis 3 revealed that SGM patients showed significantly faster initial rates of improvement (not slower, as hypothesized) than their CH peers, (*γ* = 0.316, 95% CI = 0.084, 0.547). SGM status was not a predictor of the linear time component (*γ* = 0.000, 95% CI = −0.003, 0.003), meaning that SGM patients and their CH peers progressed at roughly the same rates after the initial phase of treatment. See Table 2 for EMMs by SGM status over time and Table 3 (Model 3) for parameter estimates.

**Table 2:** Estimated marginal mean global EDE‐Q scores over treatment by sexual/gender minority status

		Sexual/gender minority			Unsure of sexual orientation			Cisgender heterosexual		
Treatment stage	Day	Mean	*SE*	95% CI	Mean	*SE*	95% CI	Mean	*SE*	95% CI
RTC admission	0	4.106	0.079	(3.950, 4.261)	3.686	0.122	(3.446, 3.925)	3.684	0.041	(3.603, 3.765)
RTC discharge	38	2.334	0.077	(2.182, 2.486)	2.294	0.124	(2.050, 2.538)	2.219	0.041	(2.139, 2.299)
PHP discharge	73	2.171	0.084	(2.006, 2.337)	2.047	0.127	(1.798, 2.295)	2.060	0.045	(1.972, 2.148)
IOP discharge	119	1.973	0.138	(1.702, 2.244)	1.733	0.199	(1.342, 2.124)	1.864	0.075	(1.717, 2.010)

*Note*: Days represent median discharge times for patients enrolled in all three levels‐of‐care.

Abbreviations: EDE‐Q, Eating Disorder Examination Questionnaire; IOP, intensive outpatient program; PHP, partial hospitalization program; RTC, residential treatment center.

**Table 3:** Progression of mixed‐effects models predicting global EDE‐Q scores over treatment by sexual/gender minority status

	Model 1 (*N* = 2818)		Model 2 (2818)		Model 3 (*N* = 2813)		Model 4^a^(*N* = 2797)	
	*γ* (*SE*)	*df*	*γ* (*SE*)	*df*	*γ* (*SE*)	*df*	*γ* (*SE*)	*df*
*Fixed effects*								
*Model for initial status (intake EDE‐Q score)*								
*Intercept* b	**2.887 (0.027)** ***	**2597**	**3.756 (0.037)** ***	**2556**	**3.684 (0.041)** ***	**2554**	**4.026 (0.086)** ***	**2527**
Initial level of care—PHP	**−0.333 (0.097)** ***	**2345**	**−0.290 (0.086)** **	2564	**−0.309 (0.086)** ***	**2560**	**−0.439 (0.084)** ***	**2522**
Initial level of care—IOP	**−0.341 (0.073)** **	**3171**	**−0.576 (0.106)** ***	2560	**−0.555 (0.106)** ***	**2556**	**−0.783 (0.109)** ***	**2529**
Initial level of care—RTC (reference)					**‐**	**‐**		
Sexual/gender minority					**0.422 (0.085)** ***	**2563**		
Unsure of sexual orientation					0.002 (0.127)	2532		
Cisgender heterosexual (reference)					**‐**	**‐**		
*Covariates (and gender moderation)*								
Sexual/gender minority male							0.195 (0.302)	2506
Sexual/gender minority female							**0.223 (0.086)** **	**2526**
Unsure of sexual orientation male							−0.456 (0.398)	2467
Unsure of sexual orientation female							0.020 (0.129)	2499
Cisgender heterosexual male							**−1.156 (0.161)** ***	**2507**
Cisgender heterosexual female (reference)							**‐**	**‐**
No history of sexual abuse							**−0.275 (0.077)** ***	**2515**
No history of other trauma							−0.052 (0.071)	2522
No history of bullying							**−0.262 (0.065)** ***	**2516**
Age 19 and older							**0.238 (0.081)** **	**2586**
Months since eating disorder onset^c^							0.073 (0.041)	2740
No prior eating disorder treatment							0.121 (0.078)	2513
Intake body mass index^c^							**0.130 (0.033)** ***	**2609**
*Model for rate of acceleration (initial slope)*				*df*		*df*		*df*
*Intercept* d			**1.387 (0.053)** ***	**1303**	**1.344 (0.059)** ***	**1329**	**1.603 (0.126)** ***	**1383**
Initial level of care—PHP			**‐0.401 (0.113)** ***	**1457**	**−0.417 (0.113)** ***	**1452**	**−0.494 (0.114)** ***	**1430**
Initial level of care—IOP			**−0.519 (0.236)** *	**2294**	**−0.522 (0.237)** *	**2281**	**−0.581 (0.239)** *	**2194**
Initial level of care—RTC (reference)					**‐**	**‐**	**‐**	**‐**
Sexual/gender minority					**0.316 (0.118)** **	**1492**		
Unsure of sexual orientation					**−**0.174 (0.182)	1249		
Cisgender heterosexual (reference)					**‐**	**‐**		
*Covariates (and gender moderation)*								
Sexual/gender minority male							−0.206 (0.435)	1423
Sexual/gender minority female							0.200 (0.124)	1466
Unsure of sexual orientation male							−0.100 (0.591)	1168
Unsure of sexual orientation female							−0.187 (0.193)	1223
Cisgender heterosexual male							**−0.538 (0.248)** *	**1344**
Cisgender heterosexual female (reference)							**‐**	**‐**
No history of sexual abuse							−0.160 (0.112)	1412
No history of other trauma							**−0.234 (0.104)** *	**1403**
No history of bullying							−0.102 (0.095)	1389
Age 19 and older							0.091 (0.118)	1498
Months since eating disorder onset^c^							0.007 (0.061)	1779
No prior eating disorder treatment							**0.241 (0.113)** *	**1427**
Intake body mass index^c^							0.067 (0.051)	1943
*Model for rate of change (phase 2 slope)*				*df*		*df*		*df*
*Intercept* e			**−0.004 (0.001)** ***	**523**	**−0.004 (0.001)** ***	**545**	**−0.005 (0.002)** **	**583**
Initial level of care—PHP			**−0.005 (0.001)** ***	**633**	**−0.005 (0.001)** ***	**633**	**−0.005 (0.001)** ***	**615**
Initial level of care—IOP			−0.004 (0.003)	1092	**−0.005 (0.003)** ***	1092	−0.003 (0.003)	1021
Initial level of care—RTC (Reference)			**‐**	**‐**	**‐**	**‐**	**‐**	**‐**
Sexual/gender minority					0.000 (0.002)	641		
Unsure of sexual orientation					−0.003 (0.003)*	476		
Cisgender heterosexual (reference)					**‐**	**‐**		
*Covariates (and gender moderation)*								
Sexual/gender minority male							−0.006 (0.006)	761
Sexual/gender minority female							0.001 (0.002)	610
Unsure of sexual orientation male							−0.002 (0.007)	397
Unsure of sexual orientation female							−0.001 (0.003)	466
Cisgender heterosexual male							0.001 (0.004)	578
Cisgender heterosexual female (reference)							**‐**	**‐**
No history of sexual abuse							0.002 (0.002)	599
No history of other trauma							−0.001 (0.002)	590
No history of bullying							0.001 (0.001)	572
Age 19 and older							0.002 (0.002)	618
Months since eating disorder onset^c^							0.000 (0.001)	722
No prior eating disorder treatment							**−0.004 (0.002)** **	**604**
Intake body mass index^c^							0.000 (0.001)	1346
*Random effects*								
Level 1	*σ* ^2^ (*SE*)		*σ* ^2^ (*SE*)		*σ* ^2^ (*SE*)		*σ* ^2^ (*SE*)	
Within‐person	**1.833(0.043)** ***		**0.213 (0.012)** ***		**0.213 (0.012)** ***		**0.214 (0.012)** ***	
Level 2	*τ* (*SE*)		*τ* (*SE*)		*τ* (*SE*)		*τ* (*SE*)	
Initial status	**1.121 (0.057)** ***		**2.344 (0.073)** ***		**2.322 (0.073)** ***		**2.126 (0.068)** ***	
Rate of acceleration (slope 1)			**2.303 (0.133)** ***		**2.289 (0.133)** ***		**2.239 (0.132)** ***	
Rate of change (slope 2)			**0.0002 (0.000)** ***		**0.0002 (0.000)** ***		**0.0002 (0.000)** ***	
*Goodness‐of‐fit statistics*								
−2 Log likelihood	23185.8		20145.9		20136.0		19876.7	
Akaike's information criterion	23189.8		20159.9		20150.0		19890.7	

*Note:* Model 1 represents the unconditional means model (with entry level‐of‐care controlled to account for sample structure), Model 2 represents the unconditional growth model (with entry level‐of‐care controlled to account for sample structure), Model 3 tests hypotheses 2a, 2b, and 3, Model 4 is confounder‐adjusted.

Abbreviations. ED, eating disorder; EDE‐Q, Eating Disorder Examination Questionnaire; IOP, intensive outpatient program; PHP, partial hospitalization program; Ref, reference; RTC, residential treatment center.

a Model 4 intercepts represent an individual who scored at the mean on the continuous variables and had the following specifications on the categorical variables: cisgender heterosexual, female, age < 19 years, had prior ED treatment, “yes” to history of sexual abuse, trauma, and bullying.

b Interpretation of Intercept for Initial Status Model is the mean admission EDE‐Q score for the patient who entered at the residential level‐of‐care.

c Standardized variables.

d Interpretation of Intercept for Rate of Acceleration Model is the average initial improvement for the patient entering the residential level‐of‐care.

e Interpretation of Intercept for Rate of Change Model is the average improvement by discharge from final level‐of‐care for the patient entering residential treatment.

*** *p* < .001.

** *p* < .01.

* *p* < .05.

**Figure 3:**
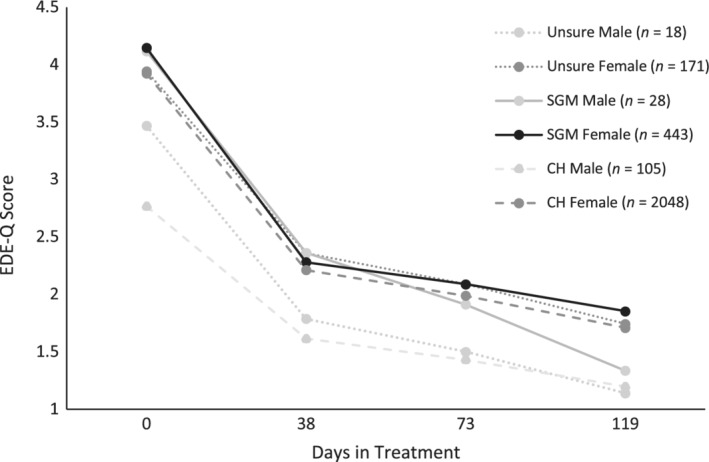
Confounder‐adjusted predicted trajectory of change in global EDE‐Q scores by gender‐identity and sexual/gender minority status over treatment through multiple levels‐of‐care (residential → partial hospital → intensive outpatient). Abbreviations: CH, cisgender heterosexual; SGM, sexual/gender minority; unsure, unsure about sexual orientation

The confounder‐adjusted model (see Table 3, Model 4), which controlled for age (<19), gender‐identity, intake BMI, months‐since‐ED onset, prior ED treatment, and all three abuse variables, found that gender‐identity was a significant effect modifier of SGM status on both ED symptom severity (Hypotheses 2a and 2b), and estimated recovery trajectories (Hypothesis 3). Compared to female CHs (the model's reference group), female SGMs showed significantly higher EDE‐Q scores at initial admission (*EMM* *Δ* = 0.223, 95% CI = 0.053, 0.393) but not higher exit scores (*EMM* *Δ* = 0.145, 95% CI = −0.152, 0.442). With covariate adjustment, female SGMs showed no different initial improvement in symptoms than female CHs, (*γ* = 0.200, 95% CI = −0.043, 0.444), and no difference in rates of improvement during the later phase of treatment (*γ* = 0.001, 95% CI = −0.003, 0.005). Compared to male CHs, male SGMs showed considerably higher EDE‐Q scores at initial admission (*EMM* *Δ* = 1.351, 95% CI = 0.691, 2.011) but not higher exit scores (*EMM* *Δ* = 0.481, 95% CI = −0.220, 1.182). While no differences emerged in rates of initial symptom improvement between male SGMs compared to female CHs (*γ* = −0.206, 95% CI = −1.060, 0.648), male CHs showed considerably slower initial improvement (*γ* = −0.538, 95% CI = −1.025, −0.052) compared to female CHs. No difference in rates of improvement during the later phase of treatment were found between male SGMs and female CHs (*γ* = −0.006, 95% CI = −0.019, 0.006) or male CHs and female CHs (*γ* = 0.001, 95% CI = −0.006, 0.008). Figure 3 shows the confounder‐adjusted trajectories of symptom scores over treatment for all sub‐groups (female CHs, male CHs, female SGMs, male SGMs, unsure females, and unsure males). Table 4 provides the corresponding EMMs for each subgroup.

**Table 4:** Confounder‐adjusted estimated marginal mean global EDE‐Q scores over treatment by gender and sexual/gender minority status

		Female										
		Sexual/gender minority				Unsure of sexual orientation				Cisgender heterosexual		
Treatment stage	Day	Mean	*SE*	95% CI		Mean	*SE*	95% CI		Mean	*SE*	95% CI
RTC admission	0	4.146	0.083	(3.984, 4.309)		3.943	0.128	(3.693, 4.193)		3.923	0.050	(3.825, 4.022)
RTC discharge	38	2.280	0.083	(2.116, 2.443)		2.361	0.133	(2.100, 2.621)		2.213	0.052	(2.112, 2.314)
PHP discharge	73	2.089	0.090	(1.912, 2.266)		2.089	0.136	(1.823, 2.355)		1.989	0.055	(1.881, 2.098)
IOP discharge	119	1.855	0.146	(1.568, 2.143)		1.745	0.215	(1.322, 2.168)		1.710	0.090	(1.534, 1.886)

		Male										
		Sexual/gender minority				Unsure of sexual orientation				Cisgender heterosexual		
Treatment stage	Day	Mean	*SE*	95% CI		Mean	*SE*	95% CI		Mean	*SE*	95% CI
RTC admission	0	4.118	0.302	(3.527, 4.710)		3.467	0.398	(2.687, 4.247)		2.767	0.162	(2.449, 3.085)
RTC discharge	38	2.362	0.312	(1.749, 2.974)		1.787	0.449	(0.906, 2.667)		1.616	0.172	(1.278, 1.955)
PHP discharge	73	1.913	0.320	(1.285, 2.540)		1.502	0.418	(0.681, 2.322)		1.432	0.175	(1.087, 1.776)
IOP discharge	119	1.336	0.497	(0.360, 2.312)		1.141	0.567	(0.028, 2.255)		1.198	0.284	(0.642, 1.755)

*Note*: Days represent median discharge times for patients enrolled in all three levels‐of‐care.

Abbreviations: EDE‐Q, Eating Disorder Examination Questionnaire; IOP, intensive outpatient program; PHP, partial hospitalization program; RTC, residential treatment center.

## 4 DISCUSSION

In light of research showing that SGMs are at a higher risk for EDs (e.g., Austin et al., 2004, 2013; Watson et al., 2017; Williamson, 2000), Minority Stress Theory (2003) has been applied to demonstrate that experiences of dehumanization and discrimination among SGMs are mediating factors predicting greater disordered eating (Brewster et al., 2019; Kamody et al., 2019; Mason & Lewis, 2016). To advance this research and previous findings about experiences of bullying/abuse among SGMs (e.g., Balsam et al., 2005; Kann et al., 2011), we used the minority stress concept to propose increased vulnerability for exposure to abuse and greater ED symptoms in a sample of ED patients receiving treatment in higher levels‐of‐care.

In support of Hypotheses 1 and 2a, we demonstrated that SGM patients have a significantly greater prevalence of experiencing varying forms of abuse and higher ED symptom severity at treatment admission compared to CH peers. Notably, the greater severity in ED symptoms may be partially explained by higher exposures to bullying and abuse found in the SGM individuals. Indeed, research demonstrates that ED patients in residential treatment with trauma have more severe ED symptoms (Scharff, Ortiz, Forrest, & Smith, 2019). The present study supports this finding in the confounder‐adjusted model showing a history of sexual abuse and bullying were significant contributors to higher symptom scores at treatment admission. In fact, when *only* adjusting for the three abuse‐related covariates (an interim confounder‐adjusted model—see Table S1), having a history of *any* of the three abuse‐related covariates uniquely contributed to greater ED symptom severity at admission. Moreover, the effect of SGM status on symptom severity at admission was reduced by a factor of 0.35 in this interim model (for comparison sake, a reduction by a factor of 0.47 is evident in the full confounder‐adjusted model), suggesting abuse and bullying history as potential mechanisms to explain why SGMs may have worse ED symptom scores than CHs at admission.

Greater ED symptom severity could also be a result of barriers to care that SGM patients have faced. A proposed model from a systematic review of the literature suggests that fear of stigma from healthcare providers may play an important role (Alencar Albuquerque et al., 2016), thus delaying diagnosis and symptom mitigating treatment. Our data show that the SGM sample was less likely to have had a prior treatment experience, and, they also had a greater median number of months since their ED onset than their CH peers (48 vs. 36 months, respectively). Barriers to equitable care for SGMs are complex and entrenched in our social mores (Dohrenwend, 2009), and they exist at both the provider level and more systemically within the US healthcare system (Buchmueller & Carpenter, 2010). Until we structurally address these matters with antidiscrimination policy that mandates healthcare provider education surrounding SGM health and expands cultural sensitivity training to include SGM allyship, health disparities like the differences shown here in ED symptom severity and abuse histories may be slow to change (Dohrenwend, 2009).

Hypothesis 2b (comparing treatment exit scores) and, relatedly, Hypothesis 3—that SGM patients would improve symptoms more slowly than CHs—were not supported. On the contrary, while both groups improved, in the unadjusted model, SGM patients experienced a significantly faster improvement in symptoms during the first treatment phase compared to CH peers. Evidence of treatment success for both SGM and CH‐identifying individuals is itself, however, an important and encouraging finding given the limited research available on treatment effectiveness in the residential treatment setting (Anderson et al., 2017). Future research should aim to test the specific components of a treatment intervention to better understand what elements make a program effective, particularly for the SGM population—in this case so much that there were no significant differences between the ED symptom severity of SGMs and CHs upon discharge. The participants in this study received a trauma‐informed (Brewerton, Alexander, & Schaefer, 2019) and compassion‐focused approach rooted in principles of Health‐At‐Every‐Size® (Tylka et al., 2014)—attributes that are all, arguably, important components of gender‐inclusive ED treatment. Additionally, SGMs received gender‐identity affirming care in that pronouns were recognized and honored, and placement in single‐sex residential treatment centers was based on gender‐identity and not sex assigned at birth or status of transition. Given the aims of this study, however, it is impossible to confirm that the gender‐inclusive aspects of the treatment environment played a role in the successful outcome for SGMs. We therefore echo the call of others (e.g., Duffy et al., 2016) for sensitivity with regards to accommodating, addressing, and valuing patients' diverse gender identities within ED treatment settings. Accordingly, we recommend future research quantitatively investigate the gender‐identity affirming aspects of the treatment milieu and associated treatment outcomes.

As with Hypothesis 2a, the effect showing SGMs' faster initial symptom improvement was attenuated after adding covariates. With confounder adjustments, the female SGMs improved only marginally faster (*p* = .11) than their female CH peers. Again, one likely explanation for this reduced effect is our accounting for abuse‐related variables. In the interim model (adjusting only for these effects), a history of sexual abuse and other trauma both made unique contributions to the initial trajectory of change. Like SGMs, those with sexual abuse and other trauma histories had significantly *faster* initial improvements in treatment. This finding is an important contribution to the research base on EDs and trauma in light of the literature proposing trauma as a potential maintenance factor of ED behaviors (Trottier, Wonderlich, Monson, Crosby, & Olmsted, 2016). The present study shows preliminary evidence suggesting that integrated trauma‐informed treatment programs have great promise for successfully targeting ED symptom change in higher levels‐of‐care. Accordingly, addressing trauma history may be a partial mechanism responsible for the early treatment success in the SGM sample. Future treatment outcomes research should aim to formally test traumatic distress as a mediator between SGM status and ED symptom change.

To further test the clinical relevance of the steeper improvement trajectory shown for the SGM sample, we conducted a post hoc examination of length of stay differences for SGMs versus CHs using a Kaplan Meier test examining “survival‐to‐discharge.” CH patients had a slightly longer median length of stay than SGM patients (49 vs. 47 days, respectively, Log‐Rank χ ^2^ = 3.54; *p* = .06). Though not a profound difference, from a clinical standpoint, 2 days at the end of residential treatment can potentially offer a meaningful shift in patient readiness to experience and flourish in the next lower level‐of‐care. This length of stay analysis adds robustness to the treatment trajectory results showing that while group differences are not vast, faster SGM symptom improvements are likely consistent with discharges due to treatment success.

This study has multiple strengths. It adds to the scant literature base on SGM patients and ED treatment. For the first time, we have data that shows SGM patients enter treatment with higher exposure rates to varying form of abuse and more severe ED symptoms. Moreover, the analyses benefitted from random effects modeling of subject‐specific symptom severity and nonlinear recovery trajectories. Our sample is relatively large for the field, and it represents a geographically diverse demographic. Nonetheless, there are limitations. The sample was comprised largely of non‐Hispanic White females who had private insurance. Therefore, it is not representative of the general population, or SGMs in particular, who are insured at lower rates than the general population (Charlton et al., 2018). The number of male‐identifying SGMs in the sample was small (*n* = 28) and though there were significant effects identified, the low *n* may have limited our ability to detect other findings related to this group. For statistical modeling purposes, we also excluded a small number of patients (*n* = 39, 1.38%) who moved from partial hospital programming (or intensive outpatient) to residential treatment centers, which could slightly bias findings toward less treatment‐resistant patients. Lastly, there may have been misclassifications on important exposure variables due to the sensitive nature of the questions. For instance, nonbinary patients may have been misgendered as female‐ or male‐identifying. Similarly, there may have been underreporting in the abuse‐related variables; research has long shown this is common with individuals who have been sexually abused (Kempe, 1978; Reitsema & Grietens, 2015).

## 5 CONCLUDING REMARKS

There are critical research‐practice gaps in understanding best care for patients with EDs (Kazdin, Fitzsimmons‐Craft, & Wilfley, 2017). Differences in how populations present and respond to treatment is understudied in the ED field (Cooper et al., 2016; Linardon, Brennan, & de la Piedad Garcia, 2016). Given the significant health disparities seen in SGMs (e.g., Hatzenbuehler & Pachankis, 2016), which our findings have underscored, it is crucial to build upon recent data showing that EDs are especially prevalent among this population (Diemer et al., 2015; Kamody et al., 2019). The present study represents an essential step toward closing the research‐practice gap by providing evidence of treatment effectiveness for this population in higher levels of ED specialty care. In future research, we suggest further advancing our knowledge by exploring potential differences in ED phenotypes that present in SGM versus CH samples. Knowing the most common ED symptom profiles among SGMs could help aid in earlier detection. Indeed, having population and clinical normative data across demographic characteristics—like SGM status—would offer a more optimal use of our existing assessment tools such as the EDE‐Q for detection of EDs in diverse subpopulations (Avila, Golden, & Aye, 2019). Reducing barriers to early intervention in the SGM population is also an important goal, especially given the treatment delay shown in this sample. The present study provides a foundation to build upon for a better understanding of normative data in a clinical sample of SGMs, as well as noting that trauma exposures may play a role in symptom exacerbation and successful treatment of EDs in SGM individuals.

## CONFLICT OF INTEREST

The authors declare no conflict of interest regarding this research. No external funding source supported the study.

## Supporting information


**Table S1** Interim confounder‐adjusted mixed‐effects model predicting global EDE‐Q scores over treatment by sexual/gender minority status and abuse‐related variables

## Data Availability

The data that support the findings of this study are available on request from the corresponding author. The data are not publicly available due to ethical restrictions.
