# Supplementary material for: Sexual and gender minority individuals report higher rates of abuse and more severe eating disorder symptoms than cisgender heterosexual individuals at admission to eating disorder treatment
Source: Int J Eat Disord. 2020 Mar 13;53(4):541–54. doi: 10.1002/eat.23257 (PMC7187146; doi:10.1002/eat.23257)
Supplement: Supplementary file 1 — Table S1 Interim confounder‐adjusted mixed‐effects model predicting global EDE‐Q scores over treatment by sexual/gender minority status and abuse‐related variables [file EAT-53-541-s001.pdf]

Supplement.

Interim confounder-adjusted mixed-effects model predicting global EDE-Q scores over treatment by sexual/gender minority status and abuse-related variables

	Model estimates (N = 2802)	
	γ (SE)	*df*
Fixed Effects		
Model for Initial Status (EDE-Q score at admission)		
Intercept†	4.27 (0.070)***	2537
Initial Level of Care – Partial Hospitalization	-0.357 (0.085)***	2547
Initial Level of Care – Intensive Outpatient	-0.575 (0.104)***	2543
Initial Level of Care – Residential Treatment (Reference)		
Sexual / Gender Minority	0.273 (0.085)***	2549
Unsure of Sexual Orientation	-0.047 (0.125)	2519
Cisgender Heterosexual (Reference)		
Abuse-related Covariates		
No History of Sexual Abuse	-0.475 (0.074)***	2533
No History of Other Trauma	-0.174 (0.070)*	2540
No History of Bullying	-0.248 (0.066)***	2540
Model for Rate of Acceleration (initial improvement)		*df*
Intercept††	1.721 (0.100)***	1378
Initial Level of Care – Partial Hospitalization	-0.451 (0.113)***	1428
Initial Level of Care – Intensive Outpatient	-0.511 (0.237)*	2236
Initial Level of Care – Residential Treatment (Reference)	--	--
Sexual / Gender Minority	0.210 (0.120)	1456
Unsure of Sexual Orientation	-0.186 (0.182)	1217
Cisgender Heterosexual (Reference)		
Abuse-related Covariates		
No History of Sexual Abuse	-0.239(0.106)*	1398
No History of Other Trauma	-0.289 (0.100)**	1388
No History of Bullying	-0.083 (0.095)	1379
Model for Rate of Change (slope during phase 2)		*df*
Intercept‡	-0.005 (0.003)**	574
Initial Level of Care – Partial Hospitalization	-0.005 (0.001)***	619
Initial Level of Care – Intensive Outpatient	-0.004 (0.003)	1073
Initial Level of Care – Residential Treatment (Reference)	--	--
Sexual / Gender Minority	0.000 (0.002)	621
Unsure of Sexual Orientation	-0.002 (0.003)	463
Cisgender Heterosexual (Reference)		--
Abuse-related Covariates		
No History of Sexual Abuse	0.001 (0.002)	593
No History of Other Trauma	-0.002 (0.002)	580
No History of Bullying	0.001 (0.001)	571
Random Effects		
Level 1	σ^2 (SE)	
Within-person	0.214 (0.012)***	
Level 2	τ (SE)	
Initial Status	2.221 (0.070)***	
Rate of Acceleration (slope 1)	2.239 (0.132)***	
Rate of Change (slope 2)	0.0002 (0.000)***	
Goodness-of-Fit Statistics		
-2 Log Likelihood	20012.9	
Akaike's Information Criterion	20026.9	

Notes. †Interpretation of Intercept for Initial Status Model is the mean admission EDE-Q score for the cisgender heterosexual patient who entered at the residential level-of-care and had a history of bullying, sexual abuse, and other trauma. ††Interpretation of Intercept for Rate of Acceleration Model is the average initial change for the patient with those same characteristics. ‡Interpretation of Intercept for Rate of Change Model is the average change by discharge from final level-of-care for the patient with those same characteristics.

Abbreviation: EDE-Q – Eating Disorder Examination Questionnaire

$^{***}p<.001$

$^{**}p<.01$

$^{*}p<.05$
